# Histologically Confirmed Testicular Metastasis Revealed by [^89^Zr]Zr-PSMA-617 PET/CT in a Patient with Biochemical Recurrence of Prostate Cancer and Negative Conventional PSMA PET/CT Imaging

**DOI:** 10.3390/diagnostics13071352

**Published:** 2023-04-05

**Authors:** Florian Rosar, Caroline Burgard, Johannes Linxweiler, Mathias Wagner, Samer Ezziddin

**Affiliations:** 1 Department of Nuclear Medicine, Saarland University, 66421 Homburg, Germany; 2 Department of Urology, Saarland University, 66421 Homburg, Germany; 3 Department of Pathology, Saarland University, 66421 Homburg, Germany

**Keywords:** prostate cancer, zirconium, PSMA, PET/CT, biochemical recurrence

## Abstract

We present an interesting image of a testicular metastasis from prostate cancer revealed by [^89^Zr]Zr-PSMA-617 PET/CT imaging in a 70-year-old man with biochemical recurrence and negative conventional [^68^Ga]Ga-PSMA-11 PET/CT imaging. This case should encourage the consideration of [^89^Zr]Zr-PSMA-617 PET/CT if conventional PSMA PET/CT imaging had failed to localize biochemical recurrence, and may remind colleagues of this rare but potential metastatic localization in this setting.

70-year-old man presented with biochemical recurrence (BCR) of prostate cancer with an increase in prostate-specific antigen (PSA) serum value to 0.65 ng/mL. After initial diagnosis of prostate cancer 3 years ago (Gleason 4 + 5 = 9, iPSA 27 ng/mL), the patient had undergone neoadjuvant androgen-deprivation-therapy (ADT) with LHRH agonists and enzalutamide, followed by robotic radical prostatectomy (pT3b pN1), followed by salvage external beam radiation of the prostate bed and pelvic lymph nodes. Most recently, the PSA increased rapidly after cycling vacations with a previously rather slow increase. For the localization of recurrence, we performed a prostate-specific membrane antigen (PSMA)-targeted positron emission tomography/computed tomography (PET/CT) using [^68^Ga]Ga-PSMA-11, which is an established imaging modality in the management of prostate cancer. [^68^Ga]Ga-PSMA-11 PET/CT (167 MBq) showed no suspicious uptake on whole-body scan 1 h post injection. Therefore, we subsequently performed a [^89^Zr]Zr-PSMA-617 PET/CT (140 MBq), which revealed an intense unequivocal focal uptake in the left testis (SUVmax 33.1 on 48 h p.i. imaging) suspicious for testicular metastasis (Figure 1, red arrow). In addition, a focal uptake was observed in a small right iliac lymph node (green arrow), suspicious for lymph node metastasis. The ultrasound of the left testis showed a corresponding hypoechogenic, hypervascularized mass, which further substantiated the suspicion of a testicular metastasis. Subsequently, a surgical resection of the metastasis (testis-sparing enucleation) was performed via a scrotal incision. Analyses including immunohistochemistry (IHC) revealed the metastatic spread of a prostatic adenocarcinoma to the left testis (Figure 2). 

PET/CT targeting the PSMA, which is a transmembrane protein overexpressed on prostate cancer cells, has revolutionized imaging of patients with prostate cancer in recent years [1,2]. PSMA PET/CT using short-lived radionuclides, as, e.g., gallium-68 (half-life: 68 min) in the form of [^68^Ga]Ga-PSMA-11 has shown a high sensitivity for tumor localization in the BCR setting [3,4]. Nevertheless, there remains an appreciable number of BCR patients without suspicions findings on [^68^Ga]Ga-PSMA-11 PET/CT. Using long-lived radionuclides, e.g., zirconium-89 (half-live: 78.4 h), in the form of [^89^Zr]Zr-PSMA-617, provides the possibility of late imaging of PET/CT up to several days post-injection, which is not possible with short-lived radionuclides, e.g., gallium-68 [5]. First clinical studies have shown that PET/CT with ^89^Zr-labeled PSMA tracers reveal lesions suspicious for prostate cancer on late images that had been missed on conventional PSMA PET/CT with short-lived PSMA tracers [6,7,8]. These previous observations, as well as the presented case, might be explained by the fact that some lesions require a longer time to internalize PSMA-targeted tracer, and, in addition, the tumor-to-background ratio increases over time due to progressive elimination of the radiopharmaceutical. Consequently, this results in a higher detection rate. Due to the longer half-life of ^89^Zr, the patients effective dose by [^89^Zr]Zr-PSMA-617 is about 2–3 times higher than with ^68^Ga [6]. This radiation exposure seems to be acceptable with respect to additional treatment-relevant information gain. Thus, ^89^Zr-based PSMA PET/CT imaging should find application in clinical routine, especially in patients with no or indetermined findings from conventional PSMA PET/CT. Further, ideally prospective studies are warranted. Prostate cancer most commonly metastasizes to the pelvic and retroperitoneal lymph nodes, the skeletal system, the lung and the liver, which is reflected by the M1a (non-regional lymph node metastases), M1b (bone metastases) and M1c (visceral metastases) substages [9]. Of note, the presence of visceral metastases reflects an aggressive tumor biology with unfavorable prognosis [9,10]. Metastases to the testes are a very rare event, although described, and are increasingly diagnosed by PSMA PET/CT [11,12,13,14,15,16,17]. When symptomatic or the single site of metastatic spread, surgical resection can be easily performed [18]. This interesting case should encourage consideration of [^89^Zr]Zr-PSMA-617 PET/CT if conventional PSMA PET/CT imaging failed localizing biochemical recurrence and may remind colleagues of this rare but potential metastatic localization in this setting.

**Figure 1:**
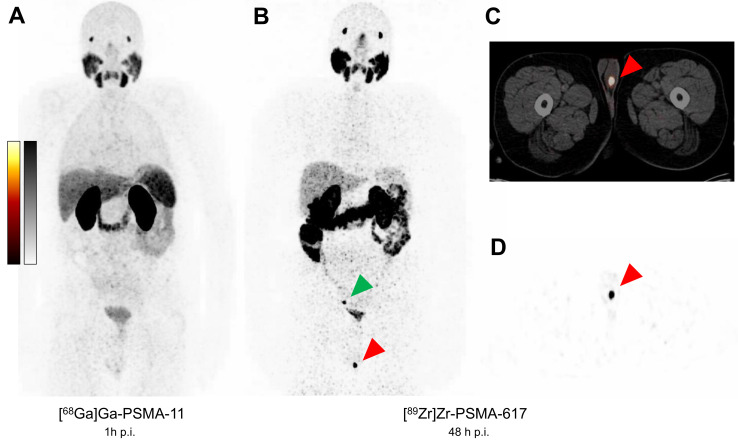
Molecular imaging. (**A**): MIP (maximum intensity projection) of [^68^Ga]Ga-PSMA-11 PET/CT (167 MBq) 1 h post injection (p.i.) without suspicious findings. (**B**): MIP of [^89^Zr]Zr-PSMA-617 PET/CT (140 MBq) 48 h p.i. and transversal slices of (**C**): PET/CT fusion and (**D**): PET showing intense focal uptake (SUV_max_ 33.1) in the left testis (red arrows) which was confirmed as testicular metastasis. Green arrow points to an intense focal uptake in the right pelvis (SUV_max_ 27.8) suspicious for an iliac lymph node metastasis. Synthesis and quality control of [^89^Zr]Zr-PSMA-617 followed published procedures [5,6]. PET/CT acquisitions were performed on a Biograph mCT 40 PET/CT scanner (Siemens Medical Solutions, Knoxville, TN, USA). The duration of PET acquisition was 3 min per bed position for ^68^Ga 1 h p.i. and was extended to 5 min per bed position for ^89^Zr 48 h p.i. PET images were reconstructed by applying an iterative 3D ordered-subset expectation maximization algorithm with 3 iterations and 24 subsets.

**Figure 2:**
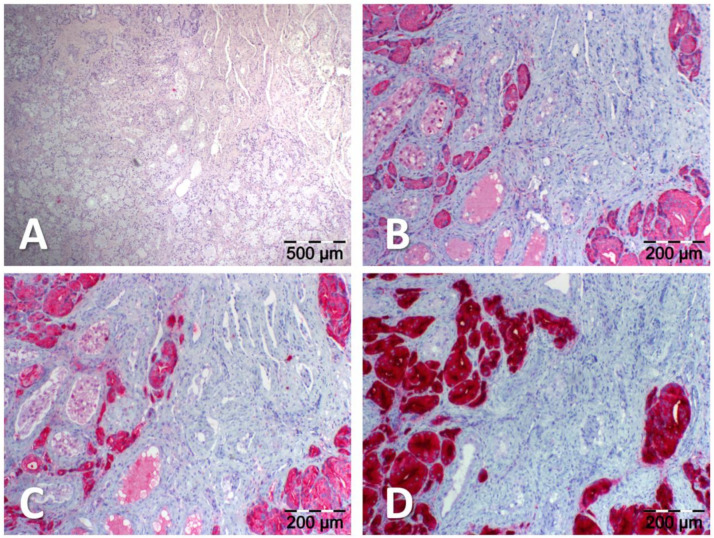
Histological findings. The site of tumor manifestation stained using hematoxylin and eosin (H&E). Lesional cells (red) were of prostatic origin as proven by the results of immunohistochemistry (IHC) with antibodies to the prostatic acid phosphatase (PAP; EC:3.1.3.2), the prostate-specific antigen (PSA; EC:3.4.21.77) or the prostate-specific membrane antigen (PSMA; EC:3.4.17.21). (**A**): H&E; (**B**): Anti-PAP IHC, Dilution: 1:5000; DAKO Agilent Technologies, Inc., Santa Clara, CA, USA; (**C**): Anti-PSA IHC, Dilution: 1:8000; DAKO; (**D**): Anti-PSMA IHC, EP192, Dilution: ready to use; Cell Marque, TM, Rocklin, CA, USA.

## Data Availability

The datasets used and analyzed in this paper are available from the corresponding author on reasonable request.

## References

[1] Ghosh A., Heston W.D.W. (2004). Tumor Target Prostate Specific Membrane Antigen (PSMA) and Its Regulation in Prostate Cancer. J. Cell. Biochem..

[2] Hofman M.S., Iravani A., Nzenza T., Murphy D.G. (2018). Advances in Urologic Imaging: Prostate-Specific Membrane Antigen Ligand PET Imaging. Urol. Clin. North. Am..

[3] Valle L., Shabsovich D., de Meerleer G., Maurer T., Murphy D.G., Nickols N.G., Vapiwala N., Calais J., Kishan A.U. (2021). Use and Impact of Positron Emission Tomography/Computed Tomography Prior to Salvage Radiation Therapy in Men with Biochemical Recurrence After Radical Prostatectomy: A Scoping Review. Eur. Urol. Oncol..

[4] Afshar-Oromieh A., da Cunha M.L., Wagner J., Haberkorn U., Debus N., Weber W., Eiber M., Holland-Letz T., Rauscher I. (2021). Performance of [^68^Ga]Ga-PSMA-11 PET/CT in Patients with Recurrent Prostate Cancer after Prostatectomy—A Multi-Centre Evaluation of 2533 Patients. Eur. J. Nucl. Med. Mol. Imaging.

[5] Privé B.M., Derks Y.H.W., Rosar F., Franssen G.M., Peters S.M.B., Khreish F., Bartholomä M., Maus S., Gotthardt M., Laverman P. (2022). ^89^Zr-Labeled PSMA Ligands for Pharmacokinetic PET Imaging and Dosimetry of PSMA-617 and PSMA-I&T: A Preclinical Evaluation and First in Man. Eur. J. Nucl. Med. Mol. Imaging.

[6] Rosar F., Schaefer-Schuler A., Bartholomä M., Maus S., Petto S., Burgard C., Privé B.M., Franssen G.M., Derks Y.H.W., Nagarajah J. (2022). [^89^Zr]Zr-PSMA-617 PET/CT in Biochemical Recurrence of Prostate Cancer: First Clinical Experience from a Pilot Study Including Biodistribution and Dose Estimates. Eur. J. Nucl. Med. Mol. Imaging.

[7] Rosar F., Bartholomä M., Maus S., Privé B.M., Khreish F., Franssen G.M., Derks Y.H.W., Nagarajah J., Ezziddin S. (2022). ^89^Zr-PSMA-617 PET/CT May Reveal Local Recurrence of Prostate Cancer Unidentified by ^68^Ga-PSMA-11 PET/CT. Clin. Nucl. Med..

[8] Dietlein F., Kobe C., Vázquez S.M., Fischer T., Endepols H., Hohberg M., Reifegerst M., Neumaier B., Schomäcker K., Drzezga A.E. (2022). An ^89^Zr-Labeled PSMA Tracer for PET/CT Imaging of Prostate Cancer Patients. J. Nucl. Med..

[9] Gandaglia G., Karakiewicz P.I., Briganti A., Passoni N.M., Schiffmann J., Trudeau V., Graefen M., Montorsi F., Sun M. (2015). Impact of the Site of Metastases on Survival in Patients with Metastatic Prostate Cancer. Eur. Urol..

[10] Ruchalski K., Kim H.J., Douek M., Raman S., Patel M., Sai V., Gutierrez A., Levine B., Fischer C., Allen-Auerbach M. (2022). Pretreatment Visceral Metastases in Castration Resistant Metastatic Prostate Cancer: Role in Prediction versus Actual Site of Disease Progression. Cancer Imaging.

[11] Gibas A., Sieczkowski M., Biernat W., Matuszewski M. (2015). Isolated Testicular Metastasis of Prostate Cancer after Radical Prostatectomy: Case Report and Literature Review. Urol. Int..

[12] Smelzo S., Mantica G., Lucianò R., Tenace N.P., De Marchi D., Pini G., Passaretti G., Losa A., Gaboardi F. (2022). Prostate Cancer Testicular Metastasis: Are They Underestimated? Case Report and Analysis of the Literature. Urologia.

[13] Gupta N., Dey S., Verma R., Belho E.S. (2020). Isolated Testicular Metastasis from Prostate Cancer Detected on Ga-68 PSMA PET/CT Scan. Nucl. Med. Mol. Imaging.

[14] da Cunha M.L., de Oliveira Rodrigues C., de Araújo M.P.L., de Freitas Junior C.H., Ferrigno R. (2018). Solitary Testicular Metastasis from Prostate Cancer. A Case Report Diagnosed by PET/CT with PSMA. Eur. J. Nucl. Med. Mol. Imaging.

[15] Ege Aktas G., Yürüt Çaloğlu V., Akdere H., Tutuğ B.B., Altun G.D. (2018). Biochemical Recurrence of Prostate Cancer Presenting as Solitary Testicular Metastasis on ^68^Ga-Labeled Prostate-Specific Membrane Antigen Ligand Positron Emission Tomography/Computed Tomography. Clin. Nucl. Med..

[16] Deeb I.A., Khdhir M.A., Bulbul M., Haidar M. (2020). Solitary Metastasis of Prostatic Adenocarcinoma to the Testicle Detected by ^68^Ga-Prostate-Specific Membrane Antigen Positron Emission Tomography/Computed Tomography. Indian J. Nucl. Med..

[17] Weiberg D., Radner H., Derlin T., Thon W.F. (2017). Early Detection of Bilateral Testicular Metastases From Prostatic Adenocarcinoma Using ^68^Ga-PSMA Ligand PET/CT. Clin. Nucl. Med..

[18] Kollitsch L., Hamann C., Knüpfer S., Meyer D., Kneissl P., Jüttner E., Osmonov D. (2020). Symptomatic testicular metastasis of acinar adenocarcinoma of the prostate. Urol. A.

